# Central Hypovolemia Detection During Environmental Stress—A Role for Artificial Intelligence?

**DOI:** 10.3389/fphys.2021.784413

**Published:** 2021-12-15

**Authors:** Björn J. P. van der Ster, Yu-Sok Kim, Berend E. Westerhof, Johannes J. van Lieshout

**Affiliations:** ^1^Department of Internal Medicine, Amsterdam University Medical Center, University of Amsterdam, Amsterdam, Netherlands; ^2^Department of Anesthesiology, Amsterdam University Medical Center, University of Amsterdam, Amsterdam, Netherlands; ^3^Laboratory for Clinical Cardiovascular Physiology, Amsterdam University Medical Center, University of Amsterdam, Amsterdam, Netherlands; ^4^Department of Internal Medicine, Medisch Centrum Leeuwarden, Leeuwarden, Netherlands; ^5^Department of Pulmonary Medicine, Amsterdam University Medical Center, Vrije Universiteit Amsterdam, Amsterdam Cardiovascular Sciences, Amsterdam, Netherlands; ^6^Medical Research Council Versus Arthritis Centre for Musculoskeletal Ageing Research, Division of Physiology, Pharmacology and Neuroscience, School of Life Sciences, The Medical School, University of Nottingham Medical School, Queen's Medical Centre, Nottingham, United Kingdom

**Keywords:** anesthesia, artificial intelligence, cardiovascular modeling, exercise, head-up tilt, hypovolemia, lower body negative pressure, POTS

## Abstract

The first step to exercise is preceded by the required assumption of the upright body position, which itself involves physical activity. The gravitational displacement of blood from the chest to the lower parts of the body elicits a fall in central blood volume (CBV), which corresponds to the fraction of thoracic blood volume directly available to the left ventricle. The reduction in CBV and stroke volume (SV) in response to postural stress, post-exercise, or to blood loss results in reduced left ventricular filling, which may manifest as orthostatic intolerance. When termination of exercise removes the leg muscle pump function, CBV is no longer maintained. The resulting imbalance between a reduced cardiac output (CO) and a still enhanced peripheral vascular conductance may provoke post-exercise hypotension (PEH). Instruments that quantify CBV are not readily available and to express which magnitude of the CBV in a healthy subject should remains difficult. In the physiological laboratory, the CBV can be modified by making use of postural stressors, such as lower body “negative” or sub-atmospheric pressure (LBNP) or passive head-up tilt (HUT), while quantifying relevant biomedical parameters of blood flow and oxygenation. Several approaches, such as wearable sensors and advanced machine-learning techniques, have been followed in an attempt to improve methodologies for better prediction of outcomes and to guide treatment in civil patients and on the battlefield. In the recent decade, efforts have been made to develop algorithms and apply artificial intelligence (AI) in the field of hemodynamic monitoring. Advances in quantifying and monitoring CBV during environmental stress from exercise to hemorrhage and understanding the analogy between postural stress and central hypovolemia during anesthesia offer great relevance for healthy subjects and clinical populations.

## Introduction

In 2021, six National Guard soldiers in officer training were hospitalized after suffering dehydration during a six-mile “ruck march” with 35-pound backpacks at a military training school in Connecticut (Koenig, 2021). In 2002, the Washington Times reported the death of a young woman after participating in the Marine Corps Marathon (Nearman, 2002). Her death was attributed to severe hyponatremia following excessive hypotonic fluid intake. Thirteen percent out of 488 runners participating in the 2002 Boston Marathon were presented with hyponatremia (serum sodium concentration <135 mmol/l); 0.6% had severe hyponatremia (<120 mmol/l). More than 70% of 716 (38% female) runners reported water loading prior to the marathon (Almond et al., 2005). The runners attempted to maintain their central blood volume (CBV) at the cost of water intoxication just trying to prevent what in Band of Brothers First Lieutenant Harry F. Welsh of 101st Airborne Division warned Pvt. Albert Blithe for: “dehydration's a soldier's worst enemy” (Ambrose, 2001). The CBV is defined as the fraction of thoracic blood volume directly available to the left ventricle. It declines in response to the assumption of the upright position, either passive head-up tilt (HUT) or active standing up with a gravitational displacement of blood away from the thorax to the lower part of the body with a fall in venous return (Van Lieshout and Secher, 1999). Capillary transmural pressure increases in the dependent parts of the body with a non-linear accumulation of blood volume in the small vessels of the leg resulting in continued filtration into the tissue spaces and a further fall in circulating volume (Smith and Ebert, 1990; Truijen et al., 2012). The hydrostatic load of 5 min of quiet standing induces a transcapillary loss of plasma volume of ~400 ml (Lundvall and Bjerkhoel, 1994). The CBV may also become reduced in response to heat stress related to heavy physical exercise or to blood loss with the development of orthostatic intolerance (Wilson et al., 2006; Crandall et al., 2008, 2019; Gonzalez-Alonso et al., 2008; Lucas et al., 2013). Hemorrhage as a leading cause of death both in the military and civilian trauma casualty settings leads to a reduction in cerebral blood flow (CBF) and aggravates postural intolerance (Convertino et al., 2007, 2008, 2011; Crandall et al., 2019). Instruments to quantify CBV are not readily available, leaving decisions on fluid management based on the traditional parameters of blood pressure (BP) and heart rate (HR) (Jacobsen et al., 1986; Vincent and De Backer, 2013; van der Ster, 2019). Although these parameters are considered prime indicators of shock, they regularly do not reflect the loss of blood or fluid until syncope is imminent (Barcroft et al., 1944; McMichael, 1944; Secher et al., 1992; Bishop et al., 1993; Wo et al., 1993; Dabrowski et al., 2000; Harms et al., 2003). There is hardly a relationship between BP and oxygen delivery (Bartels et al., 2016) whereas tissues are in need of arterial blood flow rather than arterial pressure (Jarisch, 1928; Nichols and O'Rourke, 2005). In this mini-review, a perspective on central hypovolemia in humans is presented in relation to postural stress, exercise, and during anesthesia with a focus on early detection of central hypovolemia in the physiology lab and in the operating theater.

## Postural Stress and Exercise

Obviously, in humans, the first step to exercise is preceded by the required assumption of the upright body position (Henry et al., 1951; Gauer and Thron, 1965; Wieling and van Lieshout, 1993). The act of standing itself involves physical exercise manifested by an increase in muscle sympathetic neural activity, ventilation, oxygen consumption, and carbon dioxide production (Bjurstedt et al., 1962; Wieling et al., 1996; Van Lieshout et al., 2001; Immink et al., 2006). The abrupt gravitational displacement of blood from the chest to the lower parts of the body upon standing elicits a fall in CBV and cardiac output (CO) (McMichael and Sharpey-Schafer, 1944; Harms et al., 1999, 2003). In parallel with this gravitational shift of blood, the sensitivity of the cardiac baroreflex decreases linearly, usually within 1 min (Westerhof et al., 2006; Truijen et al., 2012; Secher and Van Lieshout, 2013). Standing up creates a heart-brain hydrostatic gradient resulting in a reduction of CBF and a fall in CO by posture or cardiac disease contributes to it (Scheinberg and Stead, 1949; Hellstrøm et al., 1994; Ide et al., 1998; Pott et al., 2000; Van Lieshout et al., 2003; Bronzwaer et al., 2017b; Junejo et al., 2019, 2020; Vlastra et al., 2020; Claassen et al., 2021). Together with the acute vasodilatation in the active leg muscles, this sequence of events initiates autonomic cardiovascular reflex activity with an increase in HR and peripheral vascular resistance until an early steady state has been reached after ~2 min in the upright position with slightly reduced CO, elevated HR, and increased diastolic BP and with an impact on CBF and brain cortical oxygenation (Piorry, 1826; Hill, 1895; Sjöstrand, 1952; Gauer and Thron, 1965; Blomqvist and Stone, 1984; Bode, 1991; Levine et al., 1994; Wieling et al., 1996; Pott et al., 2000; Shoemaker et al., 2001; Van Lieshout et al., 2001; Harms et al., 2003, 2010, 2020; Immink et al., 2006). The effects of exercising in the upright vs. seated position on cardiac preload are exemplified by a lower HR during ergometer rowing than during treadmill running (Yoshiga and Higuchi, 2002). In a similar vein, the transition to the upright posture accompanying the majority of exercise modalities affects both the arterial supply to and the venous drainage from the brain (Van Lieshout et al., 2003; Dawson et al., 2004; Gisolf et al., 2004). The postural reduction in CBV and its magnitude during standing are relevant for exercise capacity since CBV determines CO and relates directly to work capacity and maximal oxygen uptake during exercise (Higginbotham et al., 1986; Van Lieshout and Secher, 1999; Dawson et al., 2007; Levine, 2008; Bada et al., 2012; Halliwill et al., 2013). The HR response to exercise not only relates to the active muscle mass but also to body position during exercise (Bevegård et al., 1960; Wang et al., 1960; Thadani and Parker, 1978; Kramer et al., 1982). In subjects presenting with what is designated as postural orthostatic tachycardia syndrome (POTS), standing upright has become an endeavor. The change of posture results in an excessive HR increment >30 with orthostatic intolerance but without orthostatic hypotension and a high HR response to a given level of exercise with reduced exercise capacity (Low et al., 1995, 1999; Joyner, 2012). POTS (Schondorf and Low, 1993) [also known as orthostatic tachycardia syndrome (Jacob et al., 1997) or orthostatic intolerance (Shannon et al., 2000)] is characterized by symptoms, such as fatigue, light-headedness, or dizziness, that come up as soon as the subject—free of orthostatic hypotension or evidence of cardiac or metabolic disease—assumes the standing position. POTS is heterogeneous in presentation and mechanisms involved are hypovolemia, deconditioning, and hyperadrenergic state with a reduced stroke volume (SV) (Masuki et al., 2007a,b; Low et al., 2009). Often a distant flu-like syndrome followed by a period of inactivity precedes POTS (Joyner, 2012). In 1871, Jacob Mendes Da Costa published the “Irritable Heart” which is considered as “a form of cardiac malady common among soldiers” (Da Costa, 1871). This concept of irritable heart was based on his observations during the Civil War with inspection, pulse rate, pulse quality, ventilatory rate, and auscultation as the measurement techniques available (Jarcho, 1959). Thomas Lewis recognized that what then was called the “effort syndrome” was, in fact, common among civilians and that of soldiers who suffered from it no less than 57% had been recruited from sedentary or light occupations before enlisting in the First World War (Lewis, 1919; Wood, 1941). The expression ‘postural tachycardia syndrome’ has replaced previous labels, such as Da Costa syndrome, soldier's heart, anxiety, exhaustion neuroses, and neurocirculatory asthenia (Oppenheimer and Rothschild, 1918; Wooley, 1976; Howell, 1985). Although psychological symptoms are common in POTS they usually are not causal (Masuki et al., 2007a). Nevertheless, heart and circulatory neurasthenia and soldier's heart continue to be considered as post-traumatic stress disorder by some (Dyde, 2011; Borges et al., 2020). Signs and symptoms of POTS resemble extreme deconditioning as observed following prolonged bed rest or spaceflight (Vernikos and Convertino, 1994; Levine et al., 1997; Gisolf et al., 2005; Joyner and Masuki, 2008) and most subjects suffering from this condition benefit from exercise training (Harms and van Lieshout, 2001; Shibata et al., 2012).

## Post-exercise Hypotension and Recovery

In 1898, Leonard Hill made evident that after exertion BP falls to normal far more rapidly than the pulse frequency and recognized that BP becomes depressed below the normal resting BP after severe muscular work (Hill, 1898). As soon as the exercise halts the leg muscle pump no longer contributes to maintaining venous return and thus CBV (Romero et al., 2017). The elevated CO quickly returns to baseline but the vasodilatation in the previously exercised muscles is rather sustained. This results in a temporary imbalance between a reduced CO and a still enhanced peripheral vascular conductance with the development of PEH. The decline in SV during recovery in the seated but not in the supine position indicates that the contribution of CO to the maintenance of arterial pressure is smaller in the standing position (Halliwill et al., 2013). Systolic BP is lower during seated and supine recovery post-exercise at 50% and 75% of VO_2peak_ vs. resting BP (Forjaz et al., 2004; Farinatti et al., 2009), and the lower peripheral resistance in the supine compared with the seated recovery position suggests resetting of the arterial baroreflex (Raine et al., 2001; White and Raven, 2014). An increased transfer function gain between diastolic BP and middle cerebral artery diastolic CBF velocity suggests a less effective dynamic cerebrovascular autoregulation in response to rapid decreases in BP during the initial 10 min of recovery from dynamic exercise (Ogoh et al., 2007). In addition, post-exercise pulmonary diffusion capacity for carbon monoxide (DLCO) is reduced with both membrane diffusion capacity and the capillary blood volume affected (Rasmussen et al., 1986; Hanel et al., 1994). Approximately 50% of the post-exercise reduction of DLCO has been attributed to a reduction in the pulmonary blood volume (Hanel et al., 1997). This, together with the reduction in venous return by loss of the muscle pump, more important in the upright vs. supine position, may be held responsible for symptoms of post-exercise orthostatic dizziness that regularly progresses to full vasovagal syncope (Holtzhausen et al., 1994; Van Lieshout et al., 2003; Takahashi et al., 2005; Roberts, 2007). A single bout of aerobic exercise is already sufficient to produce PEH, and attempts have been made to predict (Halliwill et al., 2013; Lacewell et al., 2014) and combat it (van Lieshout et al., 1992; Krediet et al., 2002, 2006). Across pre-hypertensive subjects, BP exhibits a strong positive correlation between the fall after a single session of aerobic exercise and the reduction observed after 8 weeks of aerobic training (Brito et al., 2018). A major precipitating factor for the development of PEH is prolonged exercise in the heat, resulting in hyperthermia and dehydration with bodyweight loss, and a significant reduction in CO, muscle, and skin blood flow (Gonzalez-Alonso, 1998; Hayes et al., 2000; Wilson et al., 2006; Luttrell and Halliwill, 2015; Dalmau, 2019). A recent meta-analysis suggests a larger PEH in men compared to women with an inverse association between PEH and age but a competitive half-marathon triggered PEH and a reduced cardiac baroreflex in men but not in women, leaving the physiology involved hitherto rather ill-understood (Carpio-Rivera et al., 2016; Brito et al., 2018; Mourot et al., 2020). In orthostatically intolerant athletes, recovery from postural dizziness related to PEH in the upright body position is facilitated by physical countermeasures, such as leg tensing and bending and contracting lower body muscles that engage the skeletal muscle pump to augment venous return after exercise (van Lieshout et al., 1992; Van Lieshout et al., 2001; Krediet et al., 2002, 2006). In healthy subjects, the leg-tensing maneuver lowers systemic vascular resistance with an elevation in central venous pressure (CVP), SV, and CBF. A similar effect was observed by Ray who demonstrated that in the first minute of 1-legged exercise in the upright position, CVP increased and sympathetic nerve activity became reduced (Ray, 1993). External counter measures, such as application of an impedance threshold device generating negative intrathoracic pressure to enhance venous return (Rickards et al., 2007; Convertino et al., 2012; Poh et al., 2014) or lower limb compression garments, though less practical, may reduce pre-syncopal signs and symptoms after exercise as well (Privett et al., 2010; Lacewell et al., 2014).

## Estimation of CBV

**Figure 1:**
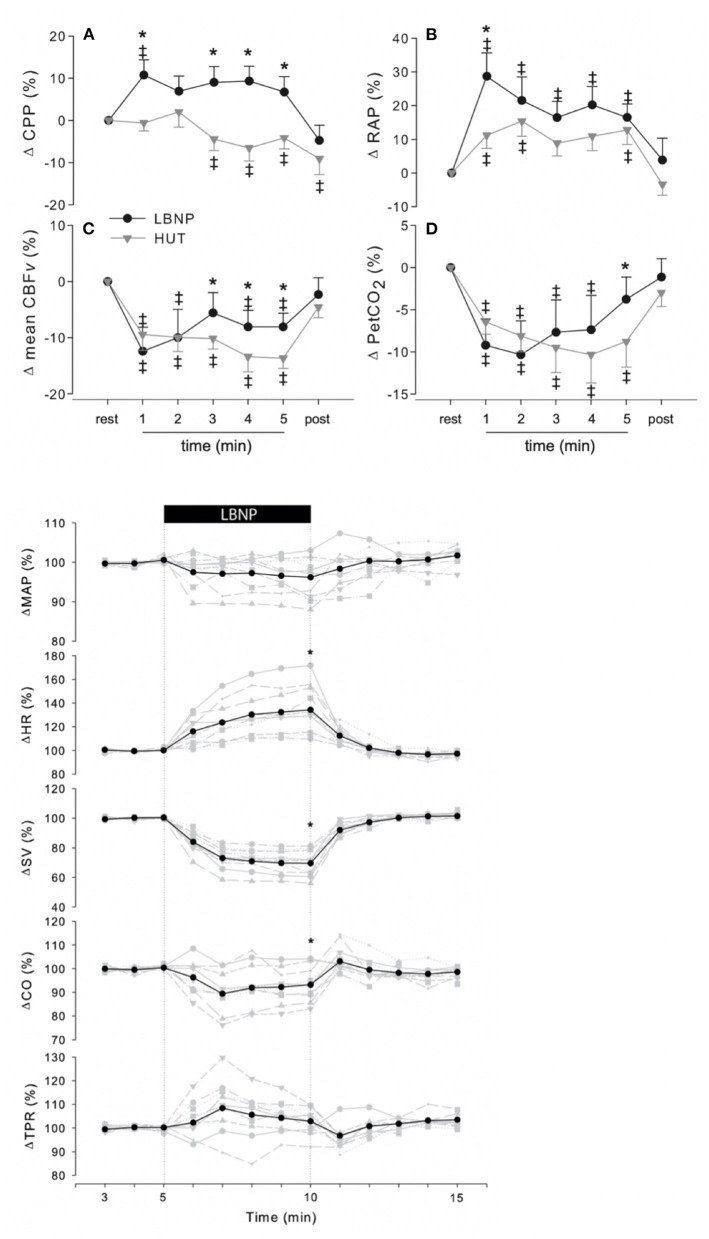
**(A–D)** Change in cerebrovascular response to 5 min LBNP−50 mmHg—(black circles) or 70◦ passive HUT (gray triangles). CPP, cerebral perfusion pressure; RAP, resistance area product; CBFv, cerebral blood flow velocity; PetCO_2_, end-tidal CO_2_; LBNP, lower body “negative” or sub-atmospheric pressure. *p* < 0.05 vs. rest; *p < 0.05 LBNP vs HUT; ‡*p* < 0.05 vs. rest. Lower panel: In 10 healthy volunteers, a total of 60 LBNP—50 mmHg—trials were performed (3 trials per subject per measurement day). Individual (gray) and averaged (black) hemodynamic responses to LBNP. Data were normalized to the last 2 min of rest. MAP, mean arterial pressure; HR, heart rate; SV, stroke volume; CO, cardiac output; TPR, total peripheral resistance; LBNP, LBNP, lower body “negative” or sub-atmospheric pressure. *p < 0.05 last 2 min of LBNP vs. last 2 min of rest. From Bronzwaer et al. (2016) and Bronzwaer et al. (2017a).

To express which magnitude the CBV in a healthy subject should have and how to quantify it remains complex (Matzen et al., 1991; Brengelmann, 2019; Dalmau, 2019; Moller and Berger, 2019; Moller et al., 2019). In the supine position, healthy humans are normovolemic in that a maximal venous oxygen saturation (S_v_O_2_) is established (Harms et al., 2003; Truijen et al., 2010). From supine to upright, S_v_O_2_ decreases and with a blood loss of ~100 ml by gravitational relocation S_v_O_2_ is reduced by 1% (Harms et al., 2003; Secher and Van Lieshout, 2005). Thoracic electrical (bio-) impedance (TI) and its reciprocal admittance offer a non-invasive index of CBV changes in humans and animals (Patterson et al., 1978; Ebert et al., 1986; Matzen et al., 1990; Perko et al., 1991; Cai et al., 2000; Ogoh et al., 2003; van Lieshout et al., 2005). In the anesthetized pig, a change in the magnitude of TI appears to be an accurate non-invasive monitor of a blood volume deficit (Krantz et al., 2000). Comparisons have been made between TI and scintigraphy with technetium-99m labeled autologous red blood cells (Crandall et al., 2008). S_v_O_2_ requires a central venous line, scintigraphy is not practical during surgery and impossible to apply with changing body position, whereas TI is non-invasive but sensitive for postural movement of the liver to be avoided by careful electrode placement. A definition of normovolemia has been proposed as the CBV that ensures optimal CO and oxygen delivery to meet metabolic demand from rest to exercise (Secher and Van Lieshout, 2005; van Lieshout et al., 2005). Evidently, this does not offer a range of reference values or cut-off points, and the next best approach is by modifying the CBV by making use of environmental stressors, while quantifying biomedical parameters for thoracic blood volume (TI), systemic (CO), regional (cerebral) blood flow, and oxygen delivery. Accordingly, expanding the CBV (Ogoh et al., 2005, 2006, 2015) or reducing it is widely applied to study the cardio- and cerebrovascular effects of simulated bleeding in healthy humans. Graded central hypovolemia is created by postural stress, either active standing, HUT or simulated orthostasis by lower body “negative” (sub-atmospheric) pressure (LBNP) with or without adding HUT to aggravate the simulated gravitational load (Stevens and Lamb, 1965; Matzen et al., 1991; Rea et al., 1991; El-Bedawi and Hainsworth, 1994; Krediet et al., 2002; Hinojosa-Laborde et al., 2011, 2014; Truijen et al., 2012; Kay and Rickards, 2015; Rosenberg et al., 2021). Qualitatively comparable to the assumption of the upright position, LBNP induces venous pooling and enhances extravasation within the leg interstitial space deliberately reducing plasma volume and CBV (Matzen et al., 1991; Hinghofer-Szalkay et al., 1992, 1996; Jørgensen et al., 1993; Truijen et al., 2012). The hemodynamic responses to graded blood loss vs. LBNP are similar with the exception that the reduction in CBF represented by transcranial Doppler CBF velocity is larger during HUT (Figure 1, upper panel). This is likely attributable to the more pronounced reduction in end-tidal CO_2_ partial pressure and the gravitational effect on cerebral perfusion pressure with HUT (Johnson et al., 2014; Bronzwaer et al., 2017a).

## Monitoring CBV in Patients

Arterial hypotension in the emergency department and during surgery promotes progressive mismatching of oxygen delivery and demand with cardiac morbidity, renal impairment, and mortality (Jones et al., 2006; Brienza et al., 2009; Wesselink et al., 2018; Wijnberge et al., 2021). There are divergent opinions on which level of BP is indicative of arterial hypotension. In fact, given a lack of a universal definition of intraoperative hypotension, the reported incidence varies substantially with the chosen threshold (Davies et al., 2020). It has been argued that such definition is an individual cut-off value and has not accurately been derived from population-based data. It is obvious that a universally accepted standard definition of hypotension would facilitate further research into this topic (Brady and Hogue, 2013; Brady et al., 2020; Etemadi and Hogue, 2020; Wijnberge et al., 2021). Traditional patient monitoring in the emergency ward and the operating room includes HR, BP, electrocardiogram, and peripheral oxygen saturation but their use as predictors for incipient central hypovolemia is rather limited. Baroreflex control of BP makes it insensitive to blood loss up to about one liter, rendering assessment of volume status by BP monitoring not possible (Harms et al., 2003; van der Ster et al., 2018b). With the progression of central hypovolemia, cardiac preload declines until the tipping point where it has become too low to maintain a sufficient CO and when the limits of vasomotor reserve available for vasoconstriction have been reached BP drops (Schondorf and Wieling, 2000; Fu et al., 2004; Schiller et al., 2017). During World War II, the observation was repeatedly made that air raid victims in London City suffering from major blood loss presented with relative bradycardia rather than the expected tachycardia (Grant and Reeve, 1941). HR does change only minimally in the early stages, and when finally becoming beyond the “normal” range, the hypovolemic shock has already developed (Secher and Van Lieshout, 2010; Schiller et al., 2017; Suresh et al., 2018). Because of these limitations, several approaches, such as wearable sensors and advanced machine-learning techniques, have been suggested in an attempt to promote more sensitive metrics for the prediction of outcomes in civil patients and on the battlefield (Secher and Van Lieshout, 2005, 2010, 2013, 2016; Rickards et al., 2007, 2008; Convertino et al., 2011, 2020a,b; Ryan et al., 2011; Nadler et al., 2014; Schlotman et al., 2019; Rashedi et al., 2021).

## Machine-Learning Based Central Hypovolemia Detection

**Figure 2:**
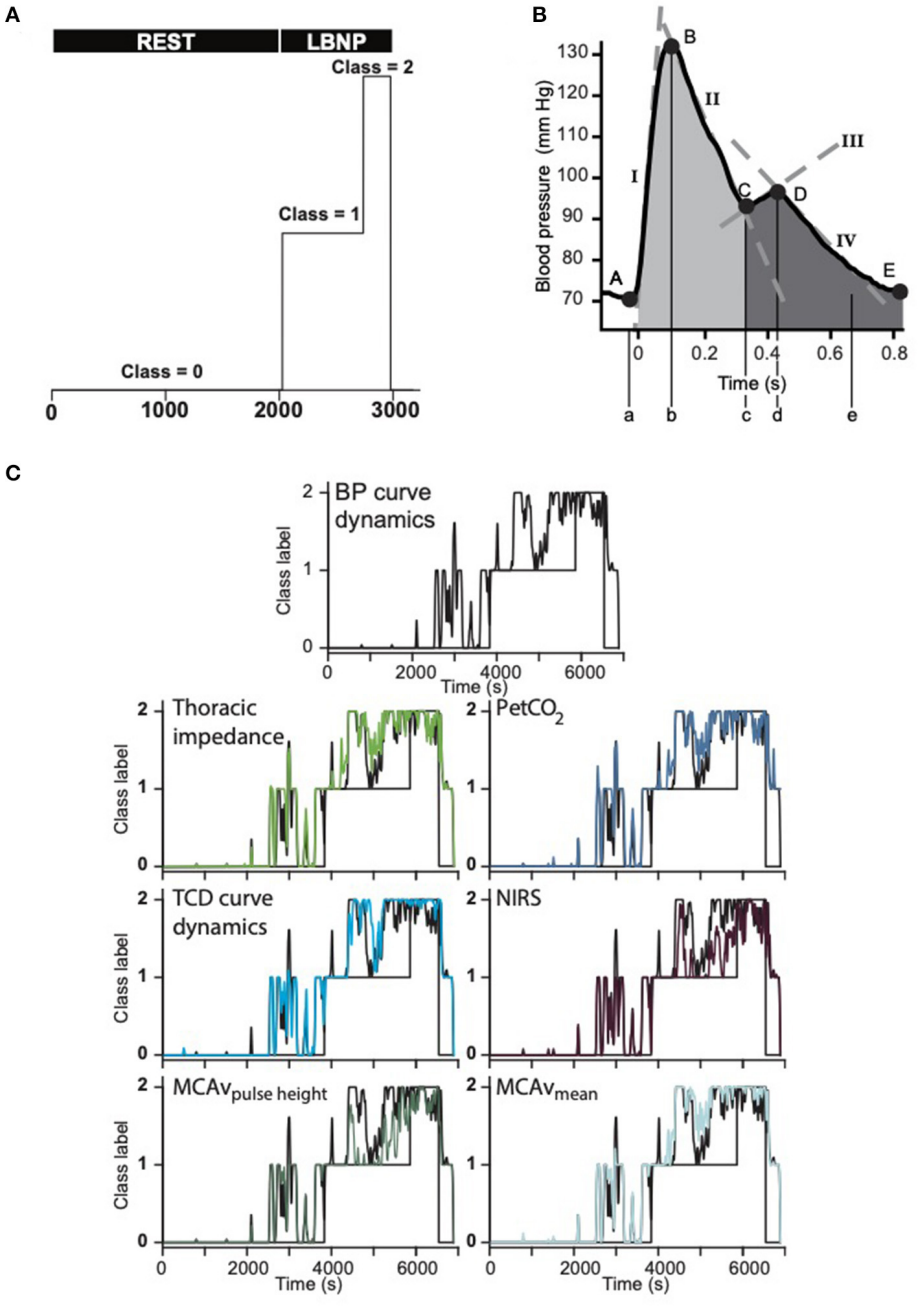
**(A)** Class definitions: baseline rest—class 0. LBNP (50 mmHg) defined as class 1, of which the last 25% as end-stage LBNP before pre-syncope (class 2). LBNP, lower body “negative” or sub-atmospheric pressure. **(B)** Single arterial pressure curve with five primary points (A–E). From these points, model parameters were estimated. Their accompanying time points are described with lower case letters. Tangent lines are described with roman numerals. Areas of interest are shaded. **(C)** In 42 (27 female) healthy subjects CBV was progressively reduced by LBNP until the onset of pre-syncope. The figure represents the output of six models compared to the blood pressure curve dynamics model (#1, top) in a single subject. Each subsequent graph shows the modulation of the addition of the annotated feature(s). In this subject the model for MCAv pulse height (bottom left) had the lowest error. Note that all model outputs increase with increasing duration of lower body negative pressure. BP, blood pressure; PetCO2, end-tidal carbon dioxide partial pressure; NIRS, near infrared spectroscopy; TCD, transcranial Doppler; MCAv, middle cerebral artery blood flow velocity; LBNP, lower body “negative” or sub-atmospheric pressure. Modified from van der Ster et al. (2018a,b).

In the last decade, efforts have been made to apply artificial intelligence (AI) and develop algorithms in the field of hemodynamic monitoring both in the operating room and on the battlefield (Convertino et al., 2008, 2011, 2020a; Rickards et al., 2014; Hatib et al., 2018; Schenk et al., 2021; Wijnberge et al., 2021). Published data on machine-learning algorithms based on arterial pressure waveform analysis are expected to play a supportive role in distinguishing normal from reduced CBV and thus left ventricular preload in healthy subjects and patients with the purpose to predict and prevent the occurrence of arterial hypotension in the anesthetized patient (Hatib et al., 2018; Connor, 2019; van der Ster et al., 2020). The earliest work in medical AI dates to the early 1970s, when the field of AI was ~15 years old. For the anesthesiologist, a monitoring system that assists in estimating the chance of developing intraoperative hypotension and predicting it would be of help (Watt et al., 1993; Mathis et al., 2018; Connor, 2019; van der Ven et al., 2020). Machine learning specifically for medicine is not new (Shortliffe, 1993; Patel et al., 2009; Convertino et al., 2011; Deo, 2015; Handelman et al., 2018), but the introduction of devices featuring models that have been trained using machine-learning algorithms has just started entering clinics. Randomized controlled trials in the field of AI-based applications of cardiovascular monitoring are as yet scarcely available (Angus, 2020; Kang et al., 2020) but progress is being made. As an example, the so-called hypotension prediction index (HPI), a machine-learning-based arterial hypotension predictive algorithm commercially available, has been proposed useful in the operating room environment (Maheshwari et al., 2020). It is based on arterial waveform features and has been claimed to predict intraoperative hypotension; the available evidence from recent clinical studies is summarized below (Davies et al., 2020; Maheshwari et al., 2020; Schneck et al., 2020; Wijnberge et al., 2020; Schenk et al., 2021). Maheshwari et al. reported a sensitivity of 88% (85–90%) and specificity of 87% (85–90%) to identify hypotensive episode 15 min in advance (area under the receiver operating characteristic curve 0.95). The fact that about 50% of the alerts were not followed by treatment was attributed to short warning time, complex treatment algorithm, or just clinicians ignoring the alert. HPI guidance did not reduce the incidence of hypotension <65 mmHg whereas that study did neither include episodes of hypotension caused by surgical manipulations (Etemadi and Hogue, 2020; Maheshwari et al., 2020). In an unblinded randomized clinical trial, patients were randomly assigned to receive either the HPI early warning system or standard care (*n* = 34 in each group), with a goal mean BP of at least 65 mmHg in both groups (Wijnberge et al., 2020). The median time-weighted average of hypotension was 0.10 mmHg [interquartile range (IQR), 0.01–0.43 mmHg)] in the intervention group vs. 0.44 mmHg (IQR, 0.23–0.72 mmHg) in the control group. The median time of hypotension per patient was 8 min (IQR, 1.3–26 min) in the intervention group vs. 33 min (IQR, 12–60 min) in the control group. In a 2-center retrospective analysis of 255 patients undergoing major surgery, the HPI predicted hypotension 5 min before a hypotensive event with a sensitivity and specificity of 85.8% (95% CI, 85.8–85.9%) and 85.8% (95% CI, 85.8–85.9%) (area under the curve, 0.926 [95% CI, 0.925–0.926]) (Davies et al., 2020). Intraoperative HPI-guided care did not reduce the time-weighted average of post-operative hypotension (Schenk et al., 2021). The warning for hypotension in these clinical studies generally appeared shorter than the 15 min previously reported in an offline validation study (Hatib et al., 2018). A reduction in CBV in the surgical patient is caused by insensible perspiration, hemorrhage, or by the accumulation of blood in the dependent parts of the body elicited or enhanced by regional anesthesia or surgery in sitting beach-chair position (Murphy et al., 2010; Larsen et al., 2014; Salazar et al., 2019). Accordingly, it represents a mimicry of the cardiovascular stress imposed by the assumption of the upright body position (Reithner et al., 1980; Secher and Van Lieshout, 2005; Hinojosa-Laborde et al., 2014; Larsen et al., 2014; Rickards et al., 2014; Schiller et al., 2017; Suresh et al., 2018). In a laboratory model of hemorrhage simulated by progressive central hypovolemia in healthy subjects by submitting them to either LBNP, HUT or both the global hypothesis tested was that AI-based methodologies may assist in monitoring and accordingly predict the progression from normo- to hypovolemia toward presyncope/cardiovascular collapse by extracting information of biomedical signals otherwise not routinely available. Figure 2, panel B, summarizes modeling of the non-invasive BP waveform (van der Ster et al., 2018b, 2020). During simulated hemorrhage in healthy subjects, volumetric parameters together with CBF velocity hemodynamics provided the most sensitive indication of the progression of central hypovolemia (Figure 2, panel C) (van der Ster et al., 2018a; van der Ster, 2019). One should realize that AI machine-learning algorithms do not entail a dynamic learning process evolving from use in clinical patient care (Schenk et al., 2021). In addition, the algorithm is unaware of the clinical situation and does neither provide any meaningful pathophysiological information. Inherently, there is no insight in the decisional process that leads to an early warning for intraoperative hypotension. Finally, it is up to clinicians to interpret and decide whether the predicted hypotensive episode can be ignored or requires intervention (Etemadi and Hogue, 2020). Another hurdle to overcome is to prove whether machine-learning-based prediction of hypotensive episodes actually does improve quality of care (Saugel et al., 2019; Etemadi and Hogue, 2020). In the laboratory model validation for larger reductions of CBV in humans is for obvious reasons not available. This constitutes a problem when realizing that in the second phase of impending shock when CBV has been reduced by about 30% a Bezold-Jarisch-like or vasovagal reflex may terminate sympathetic activity (Jarisch and Richter, 1939; Sander-Jensen et al., 1986; Campagna and Carter, 2003). Under those conditions, the HR response deviates from the traditionally expected tachycardia (Sander-Jensen et al., 1986). From experiments on cardiovascular reflex activity in humans subjected to LBNP, it has become evident that cardiovascular reflex patterns in response to a similar degree of exposure to LBNP are diverse and unpredictable among subjects, varying from a predominant effect on HR to a consistent increase in peripheral vascular resistance (Figure 1, lower panel) (Bronzwaer et al., 2016). Clinicians need hypotension predicting algorithms that operate with more precision and earlier warning. This requires novel methods better equipped to identify the variation in vasodepression and cardio-inhibition, especially in the run-up to cardiovascular collapse when compensatory mechanisms have become exhausted (Saugel et al., 2019; van Dijk et al., 2020). Future AI modeling should take the non-linear relationships between a volume loss and the cardiovascular response into account as well as the substantial inter-individual variability in how human body systems respond to environmental stress (Murray et al., 1968; Sander-Jensen et al., 1986; Bronzwaer et al., 2016, 2017a,b).

## Author Contributions

BvdS and JJvL drafted the manuscript and Y-SK and BW helped in the literature search and edited the manuscript. All authors approved the submitted version.

## Funding

This research was supported by the Danish Cardiovascular Academy and an educational grant from Edwards Lifesciences (2010B0797).

## Conflict of Interest

The authors declare that the research was conducted in the absence of any commercial or financial relationships that could be construed as a potential conflict of interest.

## Publisher's Note

All claims expressed in this article are solely those of the authors and do not necessarily represent those of their affiliated organizations, or those of the publisher, the editors and the reviewers. Any product that may be evaluated in this article, or claim that may be made by its manufacturer, is not guaranteed or endorsed by the publisher.

## Abbreviations

AI: artificial intelligence
BP: blood pressure
CBV: central blood volume
CO: cardiac output
CO_2_: carbon dioxide
CBF: cerebral blood flow
CVP: central venous pressure
DLCO: pulmonary diffusion capacity for carbon monoxide
EKG: electrocardiogram
HPI: hypotension prediction index
HR: heart rate
HUT: passive head-up tilt
LBNP: lower body ‘negative’
(sub-atmospheric) pressure
PEH: post-exercise hypotension
POTS: postural orthostatic tachycardia syndrome
SV: left ventricular stroke volume
TI: thoracic electrical (bio-) impedance
VO_2_: volume of oxygen consumption.
